# {μ-6,6′-Dimeth­oxy-2,2′-[butane-1,4-diylbis(nitrilo­methyl­idyne)]diphenolato-1:2κ^8^ *O* ^6^,*O* ^1^,*O* ^1′^,*O* ^6′^:*O* ^1^,*N*,*N*′,*O* ^1′^}tris­(nitrato-1κ^2^ *O*,*O*′)copper(II)gadolinium(III)

**DOI:** 10.1107/S1600536810014716

**Published:** 2010-04-28

**Authors:** Christopher Chan, Xiaoping Yang, Richard A. Jones, Bradley J. Holliday, Julie M. Stanley

**Affiliations:** a Department of Chemistry & Biochemistry, The University of Texas at Austin, 1 University Station A5300, Austin, TX 78712-0165, USA

## Abstract

In the title dinuclear complex, [CuGd(C_20_H_22_N_2_O_4_)(NO_3_)_3_], the Cu^II^ ion is located in the inner N_2_O_2_ cavity of the Schiff base ligand and adopts a distorted square-planar geometry. The Gd^III^ ion is ten-coordinate being bound to ten O atoms, four from the Schiff base ligand and six from three bidentate nitrate anions. The Cu^II^ and Gd^III^ ions are linked by two phenolate O atoms of the Schiff base ligand, with a separation of 3.5185 (9) Å.

## Related literature

For general background to 3*d*–4*f* bimetallic complexes, see: Sakamoto *et al.* (2001 ▶); Winpenny (1998 ▶); Yang *et al.* (2005 ▶). For related structures, see: Fei & Fang (2008 ▶); Xing *et al.* (2008 ▶).
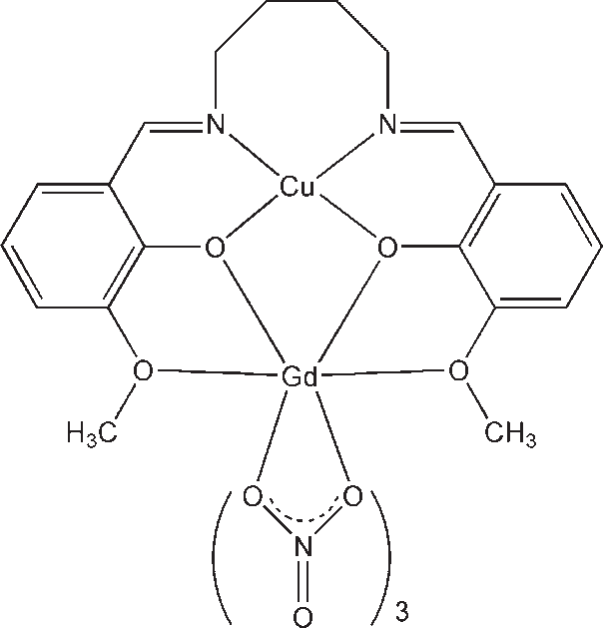


## Experimental

### 

#### Crystal data

[CuGd(C_20_H_22_N_2_O_4_)(NO_3_)_3_]
*M* *_r_* = 761.22
Monoclinic, 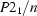

*a* = 11.795 (2) Å
*b* = 14.730 (3) Å
*c* = 14.892 (3) Å
β = 100.58 (3)°
*V* = 2543.4 (9) Å^3^
*Z* = 4
Mo *K*α radiation
μ = 3.50 mm^−1^
*T* = 223 K
0.56 × 0.13 × 0.13 mm

#### Data collection

Rigaku MiniFlexII CCD diffractometer
Absorption correction: multi-scan (*CrystalClear*; Rigaku/MSC, 2002 ▶) *T*_min_ = 0.598, *T*_max_ = 1.000
14367 measured reflections
4460 independent reflections
4090 reflections with *I* > 2σ(*I*)
*R*_int_ = 0.032

#### Refinement

*R*[*F* ^2^ > 2σ(*F* ^2^)] = 0.033
*wR*(*F* ^2^) = 0.128
*S* = 1.20
4460 reflections
362 parameters
6 restraints
H-atom parameters constrained
Δρ_max_ = 2.69 e Å^−3^
Δρ_min_ = −1.35 e Å^−3^

### 

Data collection: *CrystalClear* (Rigaku/MSC, 2002 ▶); cell refinement: *CrystalClear*; data reduction: *CrystalClear*; program(s) used to solve structure: *SHELXTL* (Sheldrick, 2008 ▶); program(s) used to refine structure: *SHELXTL*; molecular graphics: *SHELXTL*; software used to prepare material for publication: *SHELXTL*.

## Supplementary Material

Crystal structure: contains datablocks I, global. DOI: 10.1107/S1600536810014716/hy2285sup1.cif

Structure factors: contains datablocks I. DOI: 10.1107/S1600536810014716/hy2285Isup2.hkl

Additional supplementary materials:  crystallographic information; 3D view; checkCIF report

## Figures and Tables

**Table 1:** Selected bond lengths (Å)

Gd1—O1	2.581 (4)
Gd1—O2	2.336 (3)
Gd1—O3	2.418 (3)
Gd1—O4	2.568 (3)
Gd1—O5	2.472 (5)
Gd1—O6	2.458 (5)
Gd1—O8	2.472 (4)
Gd1—O9	2.428 (4)
Gd1—O11	2.431 (4)
Gd1—O12	2.520 (3)
Cu1—O2	1.941 (3)
Cu1—O3	1.940 (3)
Cu1—N1	2.004 (4)
Cu1—N2	1.960 (4)

**Fig. 1.**
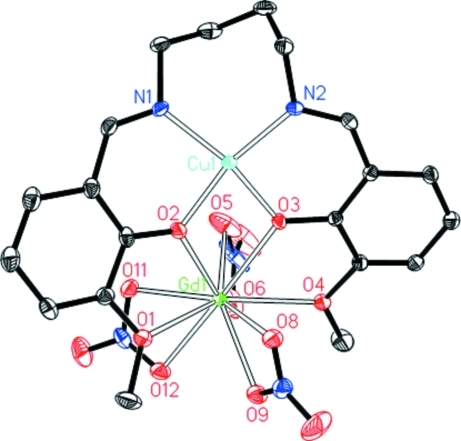
The molecular structure of the title complex, with displacement ellipsoids shown at the 40% probability level. H atoms have been excluded for clarity.

## supplementary crystallographic information

### Comment

Heteropolynuclear complexes containing d- and f-block elements are currently of great interest because of their interesting physicochemical properties and potential applications as new materials (Sakamoto *et al.*, 2001; Winpenny, 1998). Compartmental Schiff bases with two dissimilar metal-binding sites, one being specific for the d-block metal ion and another for the f-block metal ion, are classical ligands used to synthesize such heteronuclear complexes. As part of our ongoing interests in 3d–4f complexes with Schiff-base ligands (Yang *et al.*, 2005), the title complex was synthesized and its crystal structure is reported herein.

The molecular structure of the title complex is shown in Fig. 1. It has a similar structure to other two Cu–Ln complexes with the Schiff-base ligand *N*,*N*'-bis(3-methoxysalicylidene)propane-1,3-diamine (H_2_L), [CuLnL(NO_3_)_3_] [Ln = Eu^III^ (Xing *et al.*, 2008) and Tb^III^ (Fei & Fang, 2008)]. The Cu^II^ ion is coordinated by two N atoms and two O atoms from the Schiff-base ligand. The Gd^III^ ion is surrounded by ten O atoms, four from the Schiff-base ligand and six from three bidentate NO_3_^-^ anions. Cu^II^ and Gd^III^ ions are bridged by two phenolate O atoms of the Schiff-base ligand, with a separation of 3.5185 (9) Å. The average distances for the Cu—O(phenolate) and Cu—N are 1.942 Å and 1.982 Å, respectively. The Gd—O(phenolate) distance of 2.377 Å (*av.*) is shorter than the Gd—O (methoxy) distance of 2.574 Å (*av.*), no doubt reflecting the difference between ionic vs. dative bonding. The average distance for the Gd—O (nitrate) is 2.464 Å, which is comparable to those found in the literature (Fei & Fang, 2008; Xing *et al.*, 2008).

### Experimental

A mixture of the Schiff-base ligand (0.178 g, 0.5 mmol) and Cu(CH_3_CO_2_)_2_.H_2_O (0.10 g, 0.5 mmol) in EtOH (15 ml) was stirred and refluxed for 10 min. The reaction mixture was allowed to cool briefly and Gd(NO_3_)_3_.6H_2_O (0.226 g, 0.5 mmol) was added and the mixture again heated under reflux (15 min) and then filtered. Diethylether was allowed to diffuse slowly into the filtrate at room temperature and blue crystals were obtained after two weeks. The crystals were filtered off and washed with 5 ml of EtOH (yield 0.176 g, 46.32%). m. p. > 260°C (dec). ESI-MS(MeOH) m/z: 700 [M—NO_3_]^+^. IR(CH_3_OH, cm^-1^): 3429 m, 1636 s, 1473 s, 1439 w, 1362 m, 1295 w, 1227 m, 1171 w, 1099 w, 1077 m, 1013 m, 658 s. UV-VIS(CH_3_OH, 25°C) λ_max_/nm: 215, 275, 350.

### Refinement

All H atoms were positioned geometrically and refined using a riding model, with C—H = 0.94 (CH), 0.98 (CH_2_) and 0.97 (CH_3_) Å and with *U*_iso_(H) = 1.2(1.5 for methyl groups)*U*_eq_(C). The highest residual electron density was found 1.83 Å from H1A and the deepest hole 0.72 Å from Cu1.

### Crystal data

**Table:**

[CuGd(C_20_H_22_N_2_O_4_)(NO_3_)_3_]	*F*(000) = 1496
*M**_r_* = 761.22	*D*_x_ = 1.988 Mg m^−^^3^
Monoclinic, *P*2_1_/*n*	Mo *K*α radiation, λ = 0.71073 Å
Hall symbol: -P 2yn	Cell parameters from 8776 reflections
*a* = 11.795 (2) Å	θ = 1.8–31.9°
*b* = 14.730 (3) Å	µ = 3.50 mm^−^^1^
*c* = 14.892 (3) Å	*T* = 223 K
β = 100.58 (3)°	Prism, blue
*V* = 2543.4 (9) Å^3^	0.56 × 0.13 × 0.13 mm
*Z* = 4	

### Data collection

**Table:**

Rigaku MiniFlexII CCD diffractometer	4460 independent reflections
Radiation source: fine-focus sealed tube	4090 reflections with *I* > 2σ(*I*)
graphite	*R*_int_ = 0.032
Detector resolution: 13.6612 pixels mm^-1^	θ_max_ = 25.0°, θ_min_ = 2.0°
thin–slice ω scans	*h* = −14→14
Absorption correction: multi-scan (*CrystalClear*; Rigaku/MSC, 2002)	*k* = −17→17
*T*_min_ = 0.598, *T*_max_ = 1.000	*l* = −17→16
14367 measured reflections	

### Refinement

**Table:**

Refinement on *F*^2^	Secondary atom site location: difference Fourier map
Least-squares matrix: full	Hydrogen site location: inferred from neighbouring sites
*R*[*F*^2^ > 2σ(*F*^2^)] = 0.033	H-atom parameters constrained
*wR*(*F*^2^) = 0.128	*w* = 1/[σ^2^(*F*_o_^2^) + (0.0793*P*)^2^ + 1.3271*P*] where *P* = (*F*_o_^2^ + 2*F*_c_^2^)/3
*S* = 1.20	(Δ/σ)_max_ = 0.001
4460 reflections	Δρ_max_ = 2.69 e Å^−^^3^
362 parameters	Δρ_min_ = −1.35 e Å^−^^3^
6 restraints	Extinction correction: *SHELXTL* (Sheldrick, 2008)
Primary atom site location: structure-invariant direct methods	Extinction coefficient: 0.0022 (4)

### Fractional atomic coordinates and isotropic or equivalent isotropic displacement parameters (Å^2^)

**Table:**

	*x*	*y*	*z*	*U*_iso_*/*U*_eq_	
Gd1	0.261236 (19)	0.750997 (12)	0.066103 (16)	0.02180 (16)	
Cu1	0.54871 (5)	0.76924 (4)	0.03441 (4)	0.02328 (19)	
N1	0.6472 (3)	0.6645 (3)	0.0102 (3)	0.0281 (9)	
N2	0.6638 (3)	0.8666 (3)	0.0545 (3)	0.0251 (8)	
O1	0.2007 (3)	0.6647 (3)	−0.0860 (2)	0.0345 (8)	
O2	0.4094 (3)	0.6988 (2)	−0.0053 (2)	0.0305 (7)	
O3	0.4328 (3)	0.8431 (2)	0.0767 (2)	0.0275 (7)	
O4	0.2582 (3)	0.9074 (2)	0.1417 (2)	0.0337 (8)	
C1	0.0855 (5)	0.6569 (5)	−0.1376 (5)	0.0605 (18)	
H1A	0.0850	0.6743	−0.2005	0.091*	
H1B	0.0345	0.6967	−0.1116	0.091*	
H1C	0.0593	0.5947	−0.1356	0.091*	
C2	0.2860 (4)	0.6140 (3)	−0.1153 (3)	0.0294 (10)	
C3	0.2659 (5)	0.5475 (3)	−0.1817 (3)	0.0345 (11)	
H3A	0.1904	0.5355	−0.2124	0.041*	
C4	0.3593 (5)	0.4980 (4)	−0.2032 (4)	0.0406 (13)	
H4A	0.3463	0.4524	−0.2482	0.049*	
C5	0.4697 (5)	0.5162 (4)	−0.1584 (4)	0.0386 (12)	
H5A	0.5316	0.4820	−0.1721	0.046*	
C6	0.4913 (4)	0.5862 (3)	−0.0915 (3)	0.0306 (10)	
C7	0.3977 (4)	0.6343 (3)	−0.0690 (3)	0.0277 (10)	
C8	0.6085 (4)	0.5997 (3)	−0.0437 (3)	0.0326 (11)	
H8A	0.6625	0.5556	−0.0536	0.039*	
C9	0.7715 (4)	0.6524 (4)	0.0540 (4)	0.0402 (14)	
H9A	0.8203	0.6674	0.0095	0.048*	
H9B	0.7850	0.5886	0.0713	0.048*	
C10	0.8059 (5)	0.7113 (5)	0.1378 (4)	0.0426 (13)	
H10A	0.8640	0.6792	0.1820	0.051*	
H10B	0.7383	0.7215	0.1662	0.051*	
C11	0.8552 (4)	0.8039 (4)	0.1151 (4)	0.0433 (14)	
H11A	0.8623	0.8434	0.1688	0.052*	
H11B	0.9327	0.7948	0.1016	0.052*	
C12	0.7812 (5)	0.8503 (4)	0.0350 (4)	0.0338 (12)	
H12A	0.8164	0.9083	0.0230	0.041*	
H12B	0.7760	0.8123	−0.0196	0.041*	
C13	0.6435 (4)	0.9476 (3)	0.0807 (3)	0.0263 (10)	
H13A	0.7029	0.9901	0.0806	0.032*	
C14	0.5420 (4)	0.9812 (3)	0.1102 (3)	0.0238 (9)	
C15	0.5441 (4)	1.0721 (3)	0.1420 (3)	0.0286 (10)	
H15A	0.6097	1.1082	0.1417	0.034*	
C16	0.4500 (4)	1.1079 (4)	0.1733 (3)	0.0349 (11)	
H16A	0.4512	1.1684	0.1937	0.042*	
C17	0.3544 (4)	1.0541 (3)	0.1745 (3)	0.0307 (10)	
H17A	0.2913	1.0779	0.1972	0.037*	
C18	0.3500 (4)	0.9666 (3)	0.1433 (3)	0.0261 (9)	
C19	0.4434 (4)	0.9281 (3)	0.1088 (3)	0.0219 (9)	
C20	0.1675 (5)	0.9375 (5)	0.1878 (5)	0.0593 (18)	
H20A	0.2008	0.9574	0.2491	0.089*	
H20B	0.1146	0.8877	0.1912	0.089*	
H20C	0.1262	0.9875	0.1542	0.089*	
N3	0.3210 (8)	0.7319 (5)	0.2617 (4)	0.085 (3)	
O5	0.3967 (6)	0.7260 (5)	0.2116 (4)	0.0811 (19)	
O6	0.2209 (7)	0.7391 (3)	0.2219 (4)	0.0643 (17)	
O7	0.3486 (8)	0.7274 (6)	0.3444 (5)	0.135 (3)	
N4	0.1158 (4)	0.8781 (3)	−0.0484 (3)	0.0408 (11)	
O8	0.2183 (3)	0.8626 (3)	−0.0595 (2)	0.0370 (9)	
O9	0.0797 (3)	0.8273 (3)	0.0094 (3)	0.0380 (8)	
O10	0.0586 (5)	0.9393 (4)	−0.0899 (4)	0.0827 (17)	
N5	0.1497 (4)	0.5795 (3)	0.0913 (3)	0.0397 (10)	
O11	0.2586 (3)	0.5895 (3)	0.0994 (3)	0.0396 (9)	
O12	0.0899 (3)	0.6504 (3)	0.0719 (3)	0.0421 (9)	
O13	0.1069 (5)	0.5050 (3)	0.1020 (3)	0.0727 (15)	

### Atomic displacement parameters (Å^2^)

**Table:**

	*U*^11^	*U*^22^	*U*^33^	*U*^12^	*U*^13^	*U*^23^
Gd1	0.0171 (2)	0.0226 (2)	0.0268 (2)	−0.00134 (7)	0.00685 (14)	−0.00039 (7)
Cu1	0.0182 (3)	0.0237 (3)	0.0291 (3)	−0.0009 (2)	0.0074 (2)	−0.0030 (2)
N1	0.0213 (19)	0.023 (2)	0.042 (2)	0.0040 (16)	0.0126 (17)	0.0029 (18)
N2	0.0184 (18)	0.028 (2)	0.031 (2)	−0.0008 (16)	0.0101 (15)	−0.0004 (17)
O1	0.0271 (18)	0.037 (2)	0.0385 (18)	−0.0008 (16)	0.0028 (15)	−0.0079 (17)
O2	0.0229 (16)	0.0314 (18)	0.0381 (18)	−0.0031 (14)	0.0085 (14)	−0.0118 (15)
O3	0.0225 (16)	0.0221 (16)	0.0408 (18)	−0.0048 (13)	0.0132 (14)	−0.0067 (14)
O4	0.0230 (16)	0.0337 (19)	0.049 (2)	−0.0025 (15)	0.0196 (15)	−0.0092 (16)
C1	0.029 (3)	0.078 (5)	0.067 (4)	−0.001 (3)	−0.010 (3)	−0.026 (4)
C2	0.027 (2)	0.030 (3)	0.031 (2)	0.000 (2)	0.0073 (19)	0.001 (2)
C3	0.040 (3)	0.033 (3)	0.028 (2)	−0.008 (2)	0.002 (2)	−0.005 (2)
C4	0.053 (3)	0.037 (3)	0.035 (3)	−0.001 (3)	0.016 (2)	−0.009 (2)
C5	0.049 (3)	0.029 (3)	0.043 (3)	−0.002 (2)	0.022 (2)	−0.005 (2)
C6	0.035 (3)	0.024 (2)	0.035 (3)	−0.003 (2)	0.014 (2)	−0.002 (2)
C7	0.035 (3)	0.022 (2)	0.028 (2)	−0.0046 (19)	0.0119 (19)	−0.0014 (19)
C8	0.031 (2)	0.025 (2)	0.046 (3)	0.004 (2)	0.017 (2)	−0.003 (2)
C9	0.022 (3)	0.033 (3)	0.066 (4)	0.006 (2)	0.007 (3)	0.007 (3)
C10	0.026 (3)	0.058 (4)	0.043 (3)	0.004 (3)	0.004 (2)	0.010 (3)
C11	0.019 (2)	0.057 (4)	0.054 (3)	0.000 (2)	0.007 (2)	−0.013 (3)
C12	0.028 (3)	0.030 (3)	0.050 (3)	−0.005 (2)	0.024 (2)	−0.002 (2)
C13	0.026 (2)	0.027 (2)	0.027 (2)	−0.0076 (19)	0.0066 (18)	0.0012 (19)
C14	0.025 (2)	0.024 (2)	0.023 (2)	0.0006 (18)	0.0039 (17)	−0.0005 (18)
C15	0.031 (2)	0.025 (2)	0.030 (2)	−0.002 (2)	0.0064 (19)	−0.005 (2)
C16	0.034 (3)	0.029 (3)	0.041 (3)	0.002 (2)	0.004 (2)	−0.008 (2)
C17	0.026 (2)	0.031 (2)	0.038 (2)	0.004 (2)	0.0113 (19)	−0.006 (2)
C18	0.026 (2)	0.026 (2)	0.028 (2)	−0.0019 (18)	0.0096 (18)	−0.0008 (19)
C19	0.021 (2)	0.022 (2)	0.023 (2)	0.0007 (18)	0.0052 (16)	−0.0005 (18)
C20	0.041 (3)	0.065 (4)	0.083 (5)	−0.008 (3)	0.041 (3)	−0.030 (4)
N3	0.108 (7)	0.111 (5)	0.032 (3)	−0.088 (5)	−0.002 (4)	0.011 (3)
O5	0.068 (4)	0.113 (4)	0.052 (3)	−0.045 (4)	−0.015 (3)	0.027 (3)
O6	0.099 (5)	0.056 (3)	0.047 (3)	−0.031 (3)	0.037 (3)	−0.004 (2)
O7	0.149 (6)	0.203 (7)	0.047 (3)	−0.114 (5)	0.000 (4)	0.020 (4)
N4	0.038 (2)	0.036 (3)	0.048 (3)	0.001 (2)	0.008 (2)	0.006 (2)
O8	0.0287 (18)	0.035 (2)	0.049 (2)	−0.0025 (16)	0.0136 (16)	0.0100 (17)
O9	0.0300 (18)	0.041 (2)	0.044 (2)	−0.0057 (16)	0.0112 (16)	0.0052 (17)
O10	0.081 (4)	0.066 (3)	0.102 (4)	0.030 (3)	0.019 (3)	0.048 (3)
N5	0.044 (3)	0.034 (2)	0.040 (2)	−0.016 (2)	0.006 (2)	−0.001 (2)
O11	0.037 (2)	0.0297 (19)	0.053 (2)	0.0049 (16)	0.0118 (17)	0.0050 (18)
O12	0.0273 (19)	0.042 (2)	0.057 (2)	−0.0071 (17)	0.0080 (17)	0.0070 (19)
O13	0.099 (4)	0.041 (3)	0.078 (3)	−0.028 (3)	0.014 (3)	0.007 (2)

### Geometric parameters (Å, °)

**Table:**

Gd1—O1	2.581 (4)	C6—C8	1.447 (7)
Gd1—O2	2.336 (3)	C8—H8A	0.9400
Gd1—O3	2.418 (3)	C9—C10	1.512 (9)
Gd1—O4	2.568 (3)	C9—H9A	0.9800
Gd1—O5	2.472 (5)	C9—H9B	0.9800
Gd1—O6	2.458 (5)	C10—C11	1.544 (9)
Gd1—O8	2.472 (4)	C10—H10A	0.9800
Gd1—O9	2.428 (4)	C10—H10B	0.9800
Gd1—O11	2.431 (4)	C11—C12	1.507 (8)
Gd1—O12	2.520 (3)	C11—H11A	0.9800
Cu1—O2	1.941 (3)	C11—H11B	0.9800
Cu1—O3	1.940 (3)	C12—H12A	0.9800
Cu1—N1	2.004 (4)	C12—H12B	0.9800
Cu1—N2	1.960 (4)	C13—C14	1.436 (6)
N1—C8	1.276 (6)	C13—H13A	0.9400
N1—C9	1.502 (6)	C14—C19	1.398 (6)
N2—C13	1.292 (6)	C14—C15	1.419 (6)
N2—C12	1.486 (6)	C15—C16	1.385 (7)
O1—C2	1.386 (6)	C15—H15A	0.9400
O1—C1	1.437 (6)	C16—C17	1.382 (7)
O2—C7	1.332 (6)	C16—H16A	0.9400
O3—C19	1.338 (5)	C17—C18	1.367 (7)
O4—C18	1.387 (6)	C17—H17A	0.9400
O4—C20	1.443 (6)	C18—C19	1.417 (6)
C1—H1A	0.9700	C20—H20A	0.9700
C1—H1B	0.9700	C20—H20B	0.9700
C1—H1C	0.9700	C20—H20C	0.9700
C2—C3	1.381 (7)	N3—O7	1.218 (9)
C2—C7	1.403 (7)	N3—O6	1.224 (11)
C3—C4	1.406 (7)	N3—O5	1.266 (11)
C3—H3A	0.9400	N4—O10	1.224 (6)
C4—C5	1.377 (8)	N4—O8	1.270 (6)
C4—H4A	0.9400	N4—O9	1.272 (5)
C5—C6	1.424 (7)	N5—O13	1.230 (6)
C5—H5A	0.9400	N5—O12	1.264 (6)
C6—C7	1.404 (7)	N5—O11	1.277 (6)
			
O2—Gd1—O3	61.77 (11)	C2—C3—H3A	120.2
O2—Gd1—O9	132.81 (12)	C4—C3—H3A	120.2
O3—Gd1—O9	115.90 (12)	C5—C4—C3	119.9 (5)
O2—Gd1—O11	79.02 (12)	C5—C4—H4A	120.0
O3—Gd1—O11	125.29 (12)	C3—C4—H4A	120.0
O9—Gd1—O11	118.72 (12)	C4—C5—C6	120.8 (5)
O2—Gd1—O6	134.1 (2)	C4—C5—H5A	119.6
O3—Gd1—O6	106.44 (17)	C6—C5—H5A	119.6
O9—Gd1—O6	92.9 (2)	C7—C6—C5	119.0 (5)
O11—Gd1—O6	74.10 (14)	C7—C6—C8	122.4 (4)
O2—Gd1—O8	86.60 (12)	C5—C6—C8	118.4 (5)
O3—Gd1—O8	74.26 (12)	O2—C7—C2	117.9 (4)
O9—Gd1—O8	51.97 (12)	O2—C7—C6	123.2 (4)
O11—Gd1—O8	142.79 (13)	C2—C7—C6	119.0 (4)
O6—Gd1—O8	135.51 (17)	N1—C8—C6	127.7 (4)
O2—Gd1—O5	86.1 (2)	N1—C8—H8A	116.2
O3—Gd1—O5	68.18 (15)	C6—C8—H8A	116.2
O9—Gd1—O5	139.5 (2)	N1—C9—C10	112.6 (5)
O11—Gd1—O5	72.88 (18)	N1—C9—H9A	109.1
O6—Gd1—O5	50.8 (2)	C10—C9—H9A	109.1
O8—Gd1—O5	140.55 (16)	N1—C9—H9B	109.1
O2—Gd1—O12	119.60 (12)	C10—C9—H9B	109.1
O3—Gd1—O12	174.12 (12)	H9A—C9—H9B	107.8
O9—Gd1—O12	67.83 (12)	C9—C10—C11	112.4 (5)
O11—Gd1—O12	51.51 (13)	C9—C10—H10A	109.1
O6—Gd1—O12	68.33 (17)	C11—C10—H10A	109.1
O8—Gd1—O12	111.26 (12)	C9—C10—H10B	109.1
O5—Gd1—O12	105.99 (16)	C11—C10—H10B	109.1
O2—Gd1—O4	124.51 (11)	H10A—C10—H10B	107.9
O3—Gd1—O4	63.05 (10)	C12—C11—C10	112.8 (4)
O9—Gd1—O4	69.79 (12)	C12—C11—H11A	109.0
O11—Gd1—O4	141.89 (12)	C10—C11—H11A	109.0
O6—Gd1—O4	68.24 (13)	C12—C11—H11B	109.0
O8—Gd1—O4	73.61 (12)	C10—C11—H11B	109.0
O5—Gd1—O4	78.95 (19)	H11A—C11—H11B	107.8
O12—Gd1—O4	115.88 (12)	N2—C12—C11	110.1 (4)
O2—Gd1—O1	63.16 (11)	N2—C12—H12A	109.6
O3—Gd1—O1	115.29 (11)	C11—C12—H12A	109.6
O9—Gd1—O1	80.71 (12)	N2—C12—H12B	109.6
O11—Gd1—O1	71.65 (13)	C11—C12—H12B	109.6
O6—Gd1—O1	136.28 (15)	H12A—C12—H12B	108.2
O8—Gd1—O1	71.23 (12)	N2—C13—C14	128.4 (4)
O5—Gd1—O1	136.5 (2)	N2—C13—H13A	115.8
O12—Gd1—O1	69.26 (13)	C14—C13—H13A	115.8
O4—Gd1—O1	143.41 (12)	C19—C14—C15	119.7 (4)
O3—Cu1—O2	77.94 (13)	C19—C14—C13	122.7 (4)
O3—Cu1—N2	92.82 (14)	C15—C14—C13	117.6 (4)
O2—Cu1—N2	164.02 (16)	C16—C15—C14	120.5 (5)
O3—Cu1—N1	163.09 (15)	C16—C15—H15A	119.8
O2—Cu1—N1	91.16 (15)	C14—C15—H15A	119.8
N2—Cu1—N1	100.54 (16)	C17—C16—C15	119.4 (5)
O3—Cu1—Gd1	41.03 (9)	C17—C16—H16A	120.3
O2—Cu1—Gd1	38.50 (9)	C15—C16—H16A	120.3
N2—Cu1—Gd1	133.86 (11)	C18—C17—C16	121.1 (4)
N1—Cu1—Gd1	124.78 (11)	C18—C17—H17A	119.4
C8—N1—C9	113.1 (4)	C16—C17—H17A	119.4
C8—N1—Cu1	122.5 (3)	C17—C18—O4	125.4 (4)
C9—N1—Cu1	124.4 (3)	C17—C18—C19	121.2 (4)
C13—N2—C12	116.1 (4)	O4—C18—C19	113.5 (4)
C13—N2—Cu1	124.1 (3)	O3—C19—C14	123.5 (4)
C12—N2—Cu1	119.7 (3)	O3—C19—C18	118.5 (4)
C2—O1—C1	117.3 (4)	C14—C19—C18	118.1 (4)
C2—O1—Gd1	116.2 (3)	O4—C20—H20A	109.5
C1—O1—Gd1	126.2 (3)	O4—C20—H20B	109.5
C7—O2—Cu1	124.7 (3)	H20A—C20—H20B	109.5
C7—O2—Gd1	124.8 (3)	O4—C20—H20C	109.5
Cu1—O2—Gd1	110.36 (14)	H20A—C20—H20C	109.5
C19—O3—Cu1	128.0 (3)	H20B—C20—H20C	109.5
C19—O3—Gd1	124.8 (3)	O7—N3—O6	123.3 (10)
Cu1—O3—Gd1	107.17 (14)	O7—N3—O5	120.4 (10)
C18—O4—C20	116.3 (4)	O6—N3—O5	116.2 (6)
C18—O4—Gd1	119.9 (3)	N3—O5—Gd1	95.4 (5)
C20—O4—Gd1	123.7 (3)	N3—O6—Gd1	97.3 (5)
O1—C1—H1A	109.5	O10—N4—O8	121.1 (5)
O1—C1—H1B	109.5	O10—N4—O9	123.6 (5)
H1A—C1—H1B	109.5	O8—N4—O9	115.3 (4)
O1—C1—H1C	109.5	N4—O8—Gd1	95.1 (3)
H1A—C1—H1C	109.5	N4—O9—Gd1	97.2 (3)
H1B—C1—H1C	109.5	O13—N5—O12	122.9 (5)
C3—C2—O1	124.7 (4)	O13—N5—O11	121.3 (5)
C3—C2—C7	121.7 (4)	O12—N5—O11	115.8 (4)
O1—C2—C7	113.6 (4)	N5—O11—Gd1	98.3 (3)
C2—C3—C4	119.5 (5)	N5—O12—Gd1	94.4 (3)
			
O2—Gd1—Cu1—O3	159.0 (2)	O12—Gd1—O4—C18	178.8 (3)
O9—Gd1—Cu1—O3	59.78 (19)	O1—Gd1—O4—C18	−91.6 (4)
O11—Gd1—Cu1—O3	−149.68 (18)	N4—Gd1—O4—C18	−102.1 (3)
O6—Gd1—Cu1—O3	−73.1 (2)	N3—Gd1—O4—C18	101.7 (4)
O8—Gd1—Cu1—O3	67.60 (18)	O2—Gd1—O4—C20	−178.1 (4)
O5—Gd1—Cu1—O3	−81.8 (2)	O3—Gd1—O4—C20	−171.7 (5)
O12—Gd1—Cu1—O3	−168.9 (2)	O9—Gd1—O4—C20	53.2 (5)
O4—Gd1—Cu1—O3	−5.48 (18)	O11—Gd1—O4—C20	−58.0 (5)
O1—Gd1—Cu1—O3	138.55 (18)	O6—Gd1—O4—C20	−48.6 (5)
N4—Gd1—Cu1—O3	62.47 (19)	O8—Gd1—O4—C20	108.0 (5)
N3—Gd1—Cu1—O3	−76.2 (2)	O5—Gd1—O4—C20	−100.7 (5)
O3—Gd1—Cu1—O2	−159.0 (2)	O12—Gd1—O4—C20	1.9 (5)
O9—Gd1—Cu1—O2	−99.2 (2)	O1—Gd1—O4—C20	91.5 (5)
O11—Gd1—Cu1—O2	51.3 (2)	N4—Gd1—O4—C20	81.0 (5)
O6—Gd1—Cu1—O2	127.9 (2)	N3—Gd1—O4—C20	−75.2 (5)
O8—Gd1—Cu1—O2	−91.38 (19)	C1—O1—C2—C3	−9.8 (8)
O5—Gd1—Cu1—O2	119.2 (2)	Gd1—O1—C2—C3	164.2 (4)
O12—Gd1—Cu1—O2	32.1 (2)	C1—O1—C2—C7	172.0 (5)
O4—Gd1—Cu1—O2	−164.46 (19)	Gd1—O1—C2—C7	−14.0 (5)
O1—Gd1—Cu1—O2	−20.43 (19)	O1—C2—C3—C4	−177.4 (5)
N4—Gd1—Cu1—O2	−96.5 (2)	C7—C2—C3—C4	0.7 (8)
N3—Gd1—Cu1—O2	124.9 (2)	C2—C3—C4—C5	−0.3 (8)
O2—Gd1—Cu1—N2	159.0 (2)	C3—C4—C5—C6	−1.3 (8)
O3—Gd1—Cu1—N2	0.0 (2)	C4—C5—C6—C7	2.5 (8)
O9—Gd1—Cu1—N2	59.8 (2)	C4—C5—C6—C8	177.4 (5)
O11—Gd1—Cu1—N2	−149.64 (19)	Cu1—O2—C7—C2	−155.9 (3)
O6—Gd1—Cu1—N2	−73.1 (2)	Gd1—O2—C7—C2	19.0 (6)
O8—Gd1—Cu1—N2	67.64 (18)	Cu1—O2—C7—C6	24.1 (6)
O5—Gd1—Cu1—N2	−81.7 (2)	Gd1—O2—C7—C6	−160.9 (3)
O12—Gd1—Cu1—N2	−168.8 (2)	C3—C2—C7—O2	−179.4 (4)
O4—Gd1—Cu1—N2	−5.44 (18)	O1—C2—C7—O2	−1.1 (6)
O1—Gd1—Cu1—N2	138.59 (18)	C3—C2—C7—C6	0.5 (7)
N4—Gd1—Cu1—N2	62.51 (19)	O1—C2—C7—C6	178.8 (4)
N3—Gd1—Cu1—N2	−76.1 (2)	C5—C6—C7—O2	177.8 (4)
O2—Gd1—Cu1—N1	−33.5 (2)	C8—C6—C7—O2	3.2 (7)
O3—Gd1—Cu1—N1	167.5 (2)	C5—C6—C7—C2	−2.1 (7)
O9—Gd1—Cu1—N1	−132.7 (2)	C8—C6—C7—C2	−176.8 (4)
O11—Gd1—Cu1—N1	17.83 (18)	C9—N1—C8—C6	174.9 (5)
O6—Gd1—Cu1—N1	94.39 (19)	Cu1—N1—C8—C6	−3.7 (7)
O8—Gd1—Cu1—N1	−124.89 (17)	C7—C6—C8—N1	−14.0 (8)
O5—Gd1—Cu1—N1	85.7 (2)	C5—C6—C8—N1	171.3 (5)
O12—Gd1—Cu1—N1	−1.4 (2)	C8—N1—C9—C10	−162.0 (5)
O4—Gd1—Cu1—N1	162.03 (17)	Cu1—N1—C9—C10	16.6 (7)
O1—Gd1—Cu1—N1	−53.95 (17)	N1—C9—C10—C11	−92.0 (6)
N4—Gd1—Cu1—N1	−130.02 (18)	C9—C10—C11—C12	48.1 (7)
N3—Gd1—Cu1—N1	91.3 (2)	C13—N2—C12—C11	99.6 (5)
O3—Cu1—N1—C8	70.4 (7)	Cu1—N2—C12—C11	−83.1 (5)
O2—Cu1—N1—C8	21.1 (4)	C10—C11—C12—N2	59.9 (6)
N2—Cu1—N1—C8	−147.9 (4)	C12—N2—C13—C14	−175.3 (5)
Gd1—Cu1—N1—C8	41.2 (4)	Cu1—N2—C13—C14	7.6 (7)
O3—Cu1—N1—C9	−108.1 (6)	N2—C13—C14—C19	−4.1 (7)
O2—Cu1—N1—C9	−157.4 (4)	N2—C13—C14—C15	175.6 (5)
N2—Cu1—N1—C9	33.6 (4)	C19—C14—C15—C16	1.3 (7)
Gd1—Cu1—N1—C9	−137.3 (4)	C13—C14—C15—C16	−178.5 (4)
O3—Cu1—N2—C13	−4.2 (4)	C14—C15—C16—C17	0.8 (7)
O2—Cu1—N2—C13	49.9 (7)	C15—C16—C17—C18	−1.5 (7)
N1—Cu1—N2—C13	−173.7 (4)	C16—C17—C18—O4	180.0 (5)
Gd1—Cu1—N2—C13	−4.2 (5)	C16—C17—C18—C19	0.2 (7)
O3—Cu1—N2—C12	178.8 (4)	C20—O4—C18—C17	−8.6 (7)
O2—Cu1—N2—C12	−127.2 (5)	Gd1—O4—C18—C17	174.3 (4)
N1—Cu1—N2—C12	9.2 (4)	C20—O4—C18—C19	171.2 (5)
Gd1—Cu1—N2—C12	178.8 (3)	Gd1—O4—C18—C19	−5.9 (5)
O2—Gd1—O1—C2	16.4 (3)	Cu1—O3—C19—C14	5.4 (6)
O3—Gd1—O1—C2	50.7 (3)	Gd1—O3—C19—C14	−176.9 (3)
O9—Gd1—O1—C2	165.1 (3)	Cu1—O3—C19—C18	−174.7 (3)
O11—Gd1—O1—C2	−70.4 (3)	Gd1—O3—C19—C18	2.9 (5)
O6—Gd1—O1—C2	−110.5 (4)	C15—C14—C19—O3	177.3 (4)
O8—Gd1—O1—C2	112.2 (3)	C13—C14—C19—O3	−3.0 (7)
O5—Gd1—O1—C2	−33.6 (4)	C15—C14—C19—C18	−2.5 (6)
O12—Gd1—O1—C2	−125.2 (3)	C13—C14—C19—C18	177.2 (4)
O4—Gd1—O1—C2	128.9 (3)	C17—C18—C19—O3	−178.0 (4)
N4—Gd1—O1—C2	138.9 (3)	O4—C18—C19—O3	2.2 (6)
N3—Gd1—O1—C2	−73.1 (5)	C17—C18—C19—C14	1.9 (7)
O2—Gd1—O1—C1	−170.3 (5)	O4—C18—C19—C14	−178.0 (4)
O3—Gd1—O1—C1	−135.9 (5)	O7—N3—O5—Gd1	−177.0 (8)
O9—Gd1—O1—C1	−21.6 (5)	O6—N3—O5—Gd1	5.4 (7)
O11—Gd1—O1—C1	103.0 (5)	O2—Gd1—O5—N3	−166.0 (5)
O6—Gd1—O1—C1	62.8 (6)	O3—Gd1—O5—N3	132.9 (5)
O8—Gd1—O1—C1	−74.5 (5)	O9—Gd1—O5—N3	28.1 (6)
O5—Gd1—O1—C1	139.7 (5)	O11—Gd1—O5—N3	−86.3 (5)
O12—Gd1—O1—C1	48.1 (5)	O6—Gd1—O5—N3	−3.1 (4)
O4—Gd1—O1—C1	−57.8 (5)	O8—Gd1—O5—N3	114.1 (4)
N4—Gd1—O1—C1	−47.8 (5)	O12—Gd1—O5—N3	−46.3 (5)
N3—Gd1—O1—C1	100.3 (6)	O4—Gd1—O5—N3	67.7 (5)
O3—Cu1—O2—C7	161.6 (4)	O1—Gd1—O5—N3	−122.8 (5)
N2—Cu1—O2—C7	105.9 (6)	N4—Gd1—O5—N3	70.5 (5)
N1—Cu1—O2—C7	−31.4 (4)	O7—N3—O6—Gd1	177.0 (8)
Gd1—Cu1—O2—C7	175.6 (5)	O5—N3—O6—Gd1	−5.5 (8)
O3—Cu1—O2—Gd1	−13.93 (15)	O2—Gd1—O6—N3	27.4 (5)
N2—Cu1—O2—Gd1	−69.7 (6)	O3—Gd1—O6—N3	−39.0 (5)
N1—Cu1—O2—Gd1	153.03 (17)	O9—Gd1—O6—N3	−157.0 (4)
O3—Gd1—O2—C7	−163.2 (4)	O11—Gd1—O6—N3	83.9 (5)
O9—Gd1—O2—C7	−62.9 (4)	O8—Gd1—O6—N3	−123.0 (5)
O11—Gd1—O2—C7	56.3 (4)	O5—Gd1—O6—N3	3.2 (5)
O6—Gd1—O2—C7	111.0 (4)	O12—Gd1—O6—N3	138.2 (5)
O8—Gd1—O2—C7	−89.2 (4)	O4—Gd1—O6—N3	−90.2 (5)
O5—Gd1—O2—C7	129.6 (4)	O1—Gd1—O6—N3	123.4 (4)
O12—Gd1—O2—C7	23.4 (4)	N4—Gd1—O6—N3	−141.5 (4)
O4—Gd1—O2—C7	−156.6 (3)	O10—N4—O8—Gd1	171.6 (5)
O1—Gd1—O2—C7	−18.6 (3)	O9—N4—O8—Gd1	−6.9 (5)
N4—Gd1—O2—C7	−79.8 (4)	O2—Gd1—O8—N4	159.8 (3)
N3—Gd1—O2—C7	123.0 (4)	O3—Gd1—O8—N4	−138.6 (3)
O3—Gd1—O2—Cu1	12.39 (14)	O9—Gd1—O8—N4	4.2 (3)
O9—Gd1—O2—Cu1	112.66 (18)	O11—Gd1—O8—N4	92.9 (3)
O11—Gd1—O2—Cu1	−128.16 (19)	O6—Gd1—O8—N4	−41.0 (4)
O6—Gd1—O2—Cu1	−73.4 (2)	O5—Gd1—O8—N4	−120.5 (4)
O8—Gd1—O2—Cu1	86.33 (17)	O12—Gd1—O8—N4	39.2 (3)
O5—Gd1—O2—Cu1	−54.9 (2)	O4—Gd1—O8—N4	−72.7 (3)
O12—Gd1—O2—Cu1	−161.09 (14)	O1—Gd1—O8—N4	97.0 (3)
O4—Gd1—O2—Cu1	18.9 (2)	N3—Gd1—O8—N4	−77.8 (5)
O1—Gd1—O2—Cu1	157.0 (2)	O10—N4—O9—Gd1	−171.4 (5)
N4—Gd1—O2—Cu1	95.71 (18)	O8—N4—O9—Gd1	7.1 (5)
N3—Gd1—O2—Cu1	−61.4 (2)	O2—Gd1—O9—N4	−38.4 (4)
O2—Cu1—O3—C19	−168.8 (4)	O3—Gd1—O9—N4	36.1 (3)
N2—Cu1—O3—C19	−2.0 (4)	O11—Gd1—O9—N4	−140.6 (3)
N1—Cu1—O3—C19	140.3 (5)	O6—Gd1—O9—N4	146.0 (3)
Gd1—Cu1—O3—C19	178.0 (4)	O8—Gd1—O9—N4	−4.2 (3)
O2—Cu1—O3—Gd1	13.20 (14)	O5—Gd1—O9—N4	122.3 (3)
N2—Cu1—O3—Gd1	−179.97 (16)	O12—Gd1—O9—N4	−148.8 (3)
N1—Cu1—O3—Gd1	−37.6 (6)	O4—Gd1—O9—N4	80.5 (3)
O2—Gd1—O3—C19	169.8 (4)	O1—Gd1—O9—N4	−77.5 (3)
O9—Gd1—O3—C19	43.1 (4)	N3—Gd1—O9—N4	135.5 (3)
O11—Gd1—O3—C19	−140.4 (3)	O13—N5—O11—Gd1	179.4 (5)
O6—Gd1—O3—C19	−58.5 (4)	O12—N5—O11—Gd1	−0.7 (5)
O8—Gd1—O3—C19	75.1 (3)	O2—Gd1—O11—N5	−142.4 (3)
O5—Gd1—O3—C19	−92.6 (4)	O3—Gd1—O11—N5	174.3 (3)
O4—Gd1—O3—C19	−4.2 (3)	O9—Gd1—O11—N5	−9.3 (3)
O1—Gd1—O3—C19	134.9 (3)	O6—Gd1—O11—N5	75.1 (3)
N4—Gd1—O3—C19	58.2 (3)	O8—Gd1—O11—N5	−73.2 (4)
N3—Gd1—O3—C19	−73.9 (4)	O5—Gd1—O11—N5	128.2 (3)
O2—Gd1—O3—Cu1	−12.16 (13)	O12—Gd1—O11—N5	0.4 (3)
O9—Gd1—O3—Cu1	−138.80 (14)	O4—Gd1—O11—N5	84.1 (3)
O11—Gd1—O3—Cu1	37.7 (2)	O1—Gd1—O11—N5	−77.3 (3)
O6—Gd1—O3—Cu1	119.6 (2)	N4—Gd1—O11—N5	−33.3 (4)
O8—Gd1—O3—Cu1	−106.83 (17)	N3—Gd1—O11—N5	101.1 (4)
O5—Gd1—O3—Cu1	85.5 (2)	O13—N5—O12—Gd1	−179.4 (5)
O4—Gd1—O3—Cu1	173.9 (2)	O11—N5—O12—Gd1	0.7 (4)
O1—Gd1—O3—Cu1	−47.02 (19)	O2—Gd1—O12—N5	42.6 (3)
N4—Gd1—O3—Cu1	−123.76 (16)	O9—Gd1—O12—N5	170.3 (3)
N3—Gd1—O3—Cu1	104.2 (3)	O11—Gd1—O12—N5	−0.4 (3)
O2—Gd1—O4—C18	−1.2 (4)	O6—Gd1—O12—N5	−86.9 (3)
O3—Gd1—O4—C18	5.2 (3)	O8—Gd1—O12—N5	141.1 (3)
O9—Gd1—O4—C18	−130.0 (3)	O5—Gd1—O12—N5	−52.2 (4)
O11—Gd1—O4—C18	118.9 (3)	O4—Gd1—O12—N5	−137.4 (3)
O6—Gd1—O4—C18	128.2 (4)	O1—Gd1—O12—N5	82.2 (3)
O8—Gd1—O4—C18	−75.1 (3)	N4—Gd1—O12—N5	157.2 (3)
O5—Gd1—O4—C18	76.2 (3)	N3—Gd1—O12—N5	−70.6 (4)
